# Observed Reduction in Urinary Toxin Excretion With Extracorporeal Blood Oxygenation and Ozonation (EBOO) Treatment in an 88-Year-Old With Chronic Anemia: A Case Report

**DOI:** 10.7759/cureus.99948

**Published:** 2025-12-23

**Authors:** SJ Bennett, Jonathann Kuo, Serena Wang, Richard J Salway

**Affiliations:** 1 Pain Management, Hudson Health, New York, USA; 2 Chicago Medical School, Rosalind Franklin University of Health and Science, North Chicago, USA; 3 Pain Management, Extension Health, Hudson Medical Group, New York, USA; 4 Pain Management, Hudson Medical Group, New York, USA; 5 Emergency Medicine, Extension Health, New York, USA

**Keywords:** anemia, eboo, environmental toxins, extracorporeal blood oxygenation and ozonation, heavy metals, iron-deficiency anemia, mycotoxins, ozone, toxins

## Abstract

Extracorporeal blood oxygenation and ozonation (EBOO) is a hemodialysis-adjacent ex vivo filtration system. We present a case of an 88-year-old female with chronic iron-deficiency anemia with high urinary levels of mycotoxins, heavy metals, and other environmental toxins. She underwent two distinct series of three sequential EBOO treatments, with interval monitoring of her urinary toxin/creatinine ratios (µg/g) at baseline, after completion of EBOO series I, and after completion of EBOO series II. The average decline from baseline to completion of EBOO series II was 64.8% for the mycotoxins, 25.7% for the heavy metals, and 55.1% for the environmental toxins. Though there was no control for ongoing exposure, all of her toxin levels ultimately decreased except nickel. Her hemoglobin levels remained largely unchanged over the course of treatment. Thus, EBOO should be considered a potential therapy in patients with environmental toxicities that may be contributing to chronic conditions.

## Introduction

Three broad categories of toxins - mycotoxins, heavy metals, and environmental toxins - have been shown to cause and exacerbate an array of health issues. Despite their pervasiveness, there are not many treatments known to facilitate the removal of these toxins from the human body. This case report investigates extracorporeal blood oxygenation and ozonation (EBOO) as a means of removing toxins from a patient with high levels of these toxins and a 10-year history of iron-deficiency anemia.

Mycotoxins are toxic secondary metabolites produced by various species of filamentous fungi. These compounds are naturally occurring and contaminate a wide range of agricultural products, including cereals, nuts, dried fruits, and animal feeds [1]. Mycotoxin contamination is a significant global food safety concern, as these toxins are resistant to many food processing methods and can persist in the food chain [2]. Potential health consequences of mycotoxins include hepatotoxicity, nephrotoxicity, immunosuppression, carcinogenicity, and reproductive toxicity [3]. Due to their stability and ubiquity, mycotoxins represent a persistent challenge for food safety and public health, with the Food and Agriculture Organization reporting about 25% of the global food output to be contaminated by mycotoxins [1, 4].

Heavy metals are naturally occurring elements that in medical practice commonly refer to metals such as lead, mercury, cadmium, arsenic, and chromium [5-6]. These elements are notable for their toxicity, environmental persistence, and ability to bioaccumulate in living organisms, including humans [6-10]. Human exposure occurs primarily through ingestion, inhalation, or absorption via the gastrointestinal tract or skin [5-10]. Heavy metals can cause significant organ damage, neurocognitive impairment, and increased cancer risk through their ability to disrupt cellular processes and their persistence in both the environment and biological tissues [5-10].

Environmental toxins refer broadly to pesticides, phthalates, parabens, acrylics, alkyl phenols, and volatile organic compounds (VOCs). Specifically, pesticides are chemical substances, either of natural or synthetic origin, that are widely used in agriculture to reduce losses from pests. However, pesticides can be toxic to non-target organisms, persist in the environment, and bioaccumulate, leading to potential adverse effects on ecosystems and human health [11-13]. VOCs are a group of organic chemicals characterized by their high vapor pressure at room temperature, which allows them to easily evaporate into the atmosphere. They can originate from activities such as the use of pesticides, solvents, fuels, and industrial processes. VOCs are significant contributors to air pollution and are associated with adverse health effects such as respiratory irritation, neurotoxicity, and are carcinogenic [14-16].

Studies in animal models have shown that mycotoxins have been shown to cause anemia most notably via direct hemolytic effects, impaired erythropoiesis, and interference with iron metabolism [17-18]. For example, one specific class of mycotoxins, ochratoxin, can induce a hypochromic microcytic anemia of the iron-deficiency type, primarily by reducing serum iron and transferrin saturation [19]. This suggests that some mycotoxins can impair iron utilization and absorption, contributing to anemia. Mycotoxins may also induce ferroptosis, an iron-dependent form of cell death, further contributing to erythrocyte loss and anemia [20].

Similar to mycotoxins, heavy metals may exacerbate anemia. Heavy metals disrupt erythropoiesis, induce hemolysis, interfere with iron metabolism, and alter the homeostasis of essential trace elements [21-22]. Epidemiological and mechanistic studies demonstrate that exposure to many heavy metals, such as lead, cadmium, arsenic, copper, and nickel, is associated with lower hemoglobin, mean corpuscular volume (MCV), and mean corpuscular hemoglobin concentration (MCHC), leading to microcytic, hypochromic anemia, particularly in children and females [22-24].

Additionally, mouse models have shown that pesticides can cause long-lasting bone marrow suppression, depletion of hematopoietic progenitors, as well as aplastic anemia [25]. Additionally, a UK study showed that environmental air pollutants are associated with increased risk of iron-deficiency anemia [26]. Thus, environmental toxins are also associated with an increased risk of anemia through multiple mechanisms.

Humans are exposed to mycotoxins primarily through the ingestion of contaminated food, especially cereals, grains, nuts, dried fruits, and animal products, as well as inhalation of airborne mycotoxins, and even dermal contact [1-2]. Exposure to heavy metals occurs through similar methods, with ingestion of contaminated food and drinking water being the major route; inhalation exposure is significant in occupational settings [5,7,9]. Environmental toxins also make their way into humans through exposure to food and the environment. A 2023 study showed that 91.8% of people in the United States are exposed to unsafe levels of fine particulate matter through air pollution, and a 2021 study showed that 32% of the world’s population may exceed the acceptable pesticide intake [27-28]. Thus, these toxins are found nearly everywhere, and the great majority of the world’s population has been exposed to toxins at some point or another. Despite this, there is a lack of treatment options for removing toxins.

Management of high mycotoxin levels is primarily supportive and focuses on preventing further exposure and enhancing natural methods of elimination [29]. Heavy metal exposure is primarily removed from the body through chelation therapy. However, chelation therapy is only indicated for patients with severe clinical toxicity [30-31]. Environmental pollutant toxicity can potentially be mitigated by manipulating the gut microbiota with probiotics, prebiotics, or fecal microbiota transplantation, though clinical application remains investigational [32]. Thus, methods to remove toxins are primarily by encouraging the body’s natural detoxification systems and providing supportive options unless toxicity is in force. There is a lack of clinical information regarding ways to treat high levels of toxins, and there does not seem to be one method of removing multiple types of toxins at one time.

This case report studies EBOO as a potential solution to human toxicities. EBOO is a technique in which a patient’s blood is circulated outside their body and exposed to a mixture of oxygen and ozone before being returned to the patient. The process evolved from autohemotherapy (AHT) as a means to treat conditions such as severe peripheral arterial disease, coronary disease, cholesterol embolism, severe dyslipidemia, atherosclerotic vasculopathy, Madelung disease, sudden deafness of vascular origin, and macular degeneration [33-36].

EBOO functions similarly to hemodialysis, utilizing an ex vivo filtration system. When blood is exposed ex vivo to a controlled mixture of ozone and oxygen, ozone reacts rapidly with various blood components, such as antioxidants, lipids, and proteins, generating reactive oxygen species (ROS) and lipid oxidation products (LOPs) that act as secondary messengers; these molecular changes can transiently increase oxidative stress, which may stimulate endogenous antioxidant defenses and modulate immune and inflammatory responses [37-39].

EBOO is performed in approximately one hour, treating up to 4800 mL of heparinized blood at a time [33]. EBOO has been explored primarily in ex vivo and non-human research settings for potential therapeutic effects and is thus vastly understudied in toxin removal. There is also experimental investigation into ozone-AHT’s immunomodulatory effects in sepsis models, but this remains preclinical and not an established indication [40-41].

The present case highlights EBOO as a potential treatment for high levels of mycotoxins, heavy metals, and environmental toxicities as contributors to iron-deficiency anemia, highlighting the necessity of a case-specific strategy in addressing ubiquitous health problems and chronic conditions.

## Case presentation

An 88-year-old female with chronic anemia presented to the clinic for heavy metal toxicity as a potential contributor to bone marrow suppression and anemia. Previous testing indicated high levels of lead, mercury, and aluminum. She reported living in an old building with original wiring and plumbing from the 1930s and thus being constantly exposed to heavy metals, mold, and other toxins. She had been anemic for about 10 years, previously managed with pills and herbal iron supplements, and more recently managed with iron infusions. Previous providers ruled out chronic blood loss as well as systemic illness. She experienced reduced exercise tolerance, unable to walk more than 10 feet without being out of breath. She was otherwise systemically well.

Lab tests

The patient underwent blood and urine lab testing through Vibrant America Clinical Laboratory, which set normal ranges for all tests based on the National Health and Nutrition Examination Survey (NHANES) and other data from 1000 apparently healthy, unprovoked, unmedicated, and unsupplemented individuals.

Comprehensive metabolic panel and hormone panels were within normal ranges. Of note in her CBC was a hemoglobin level of 7.4 g/dL, previously as low as 4.8 g/dL. Other values, such as low hematocrit, MCV, MCH, and MCHC, were all consistent with a diagnosis of a microcytic, hypochromic anemia. Platelet counts and the remainder of the CBC were normal at this time, confirming a diagnosis of iron-deficiency anemia. Also tested were a variety of mycotoxins, heavy metals, and environmental toxins.

Also tested were a variety of mycotoxins, heavy metals, and environmental toxins. 2-Hydroxyisobutyric Acid (2HIB), an environmental toxin, as well as four notable mycotoxins, Chaetoglobosin A (CHA), Enniatin B1(ENN B1), Nivalenol (NIV), and Zearalenone (ZEN), were noted to have greater than 95th percentile exposure to the respective toxin. Suboptimal toxins, corresponding to the 75th through 95th percentile exposure, included three environmental toxins, 2,2-bis(4-Chlorophenyl) acetic acid (DDA), Diethyldithiophosphate (DEDTP), and N-Acetyl (2, Hydroxypropyl) Cysteine (NAHP), four heavy metals, barium, cadmium, lead, and nickel, as well as three mycotoxins, Aflatoxin G1, Aflatoxin M1, and Dihydrocitrinone.

Treatment plan and timeline

To improve her anemia, the patient was recommended to take over-the-counter oral iron supplements, specifically ferrous gluconate or ferrous sulfate, at a dosage of 325mg twice daily to address the severe anemia. She was also advised to start taking B12 and folate supplements to support overall blood health and potentially improve anemia. It was also recommended to consult with her hematologist about increasing the dosage of her iron infusions to enhance effectiveness.

For her high toxin and heavy metal levels, a series of three EBOO procedures was planned. As per clinic protocol, one EBOO series contains three EBOO treatments, as the EBOO processes around 2 liters of blood in one treatment, so three consecutive treatments assume that nearly all of the patient’s blood, about 5 L (4-5 L for females, 5-6 L for males), has been processed by completion of the series. The patient’s baseline labs occurred on December 19th, 2024. The EBOO series was to occur over a few-week period, with labs immediately after the third one of the series, while she was still present for her appointment. The three EBOOs took place on February 13, 18, and 27, 2025.

Next, a check-in appointment occurred on April 14, 2025, about a month after the EBOO series. The patient stated she was feeling a little bit better, and her labs noted some improvements in toxin levels. A second series of three EBOOs occurred on April 1, 10, and 29, 2025, again with labs immediately after the third one. The patient followed up again on June 25, 2025, and reported that she was feeling more tired than after the first series, so it was decided between the patient and provider that treatment was instead going to focus on her anemia with her hematologist.

**Figure 1:**
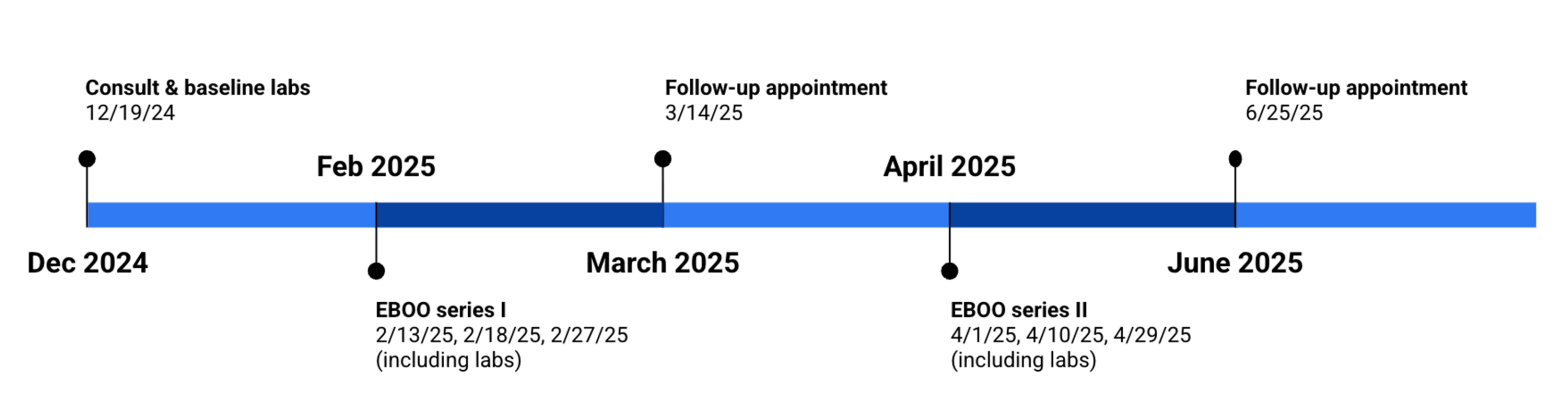
Timeline of events EBOO: Extracorporeal Blood Oxygenation and Ozonation

A succinct timeline of appointments and lab work is displayed in Figure 1.

Results

**Figure 2:**
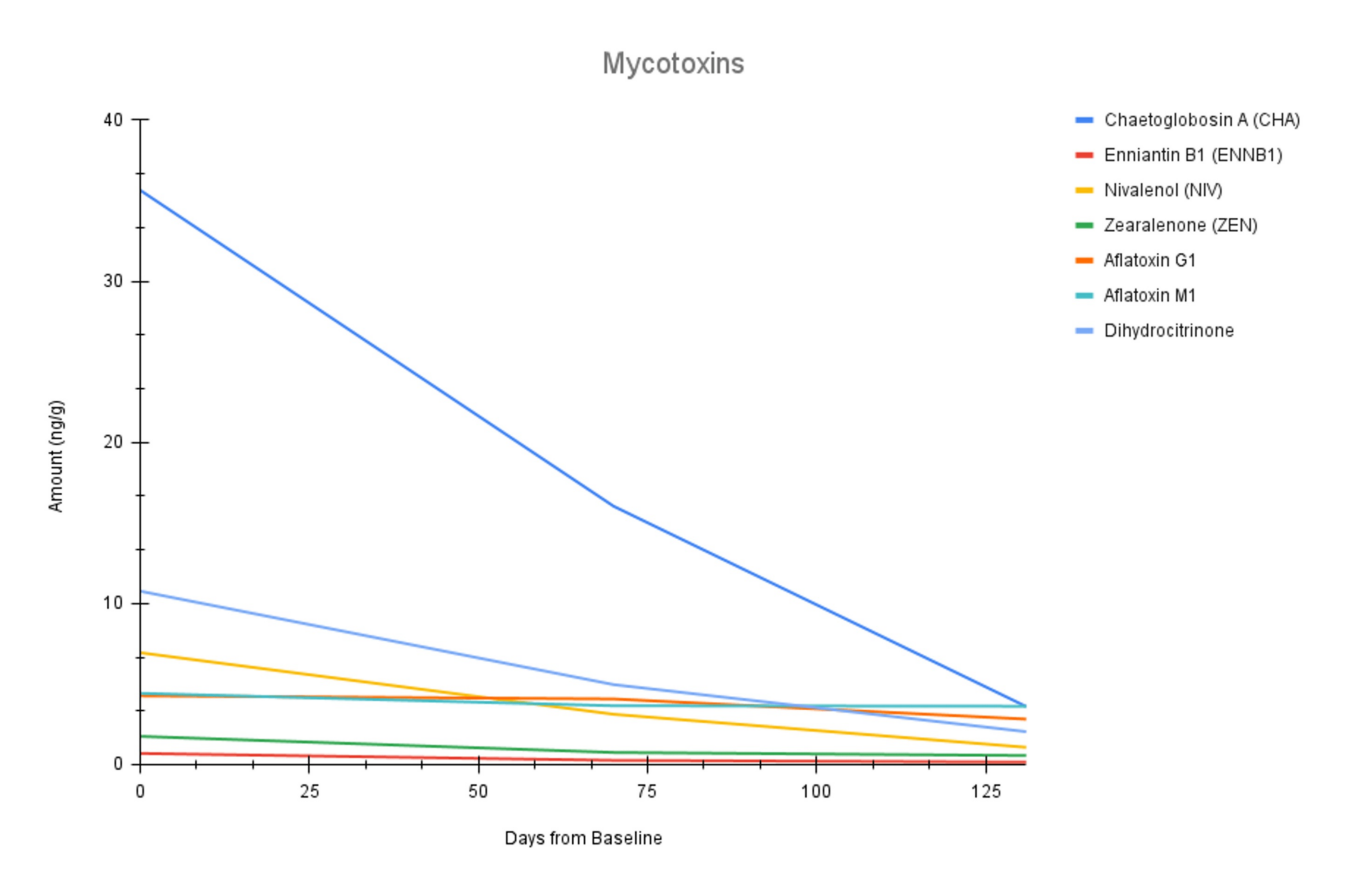
Line chart of the seven high and suboptimal mycotoxins over three time points: baseline (day 0), after completion of EBOO series I (day 70), and after completion of EBOO series II (day 131). EBOO: Extracorporeal Blood Oxygenation and Ozonation

**Figure 3:**
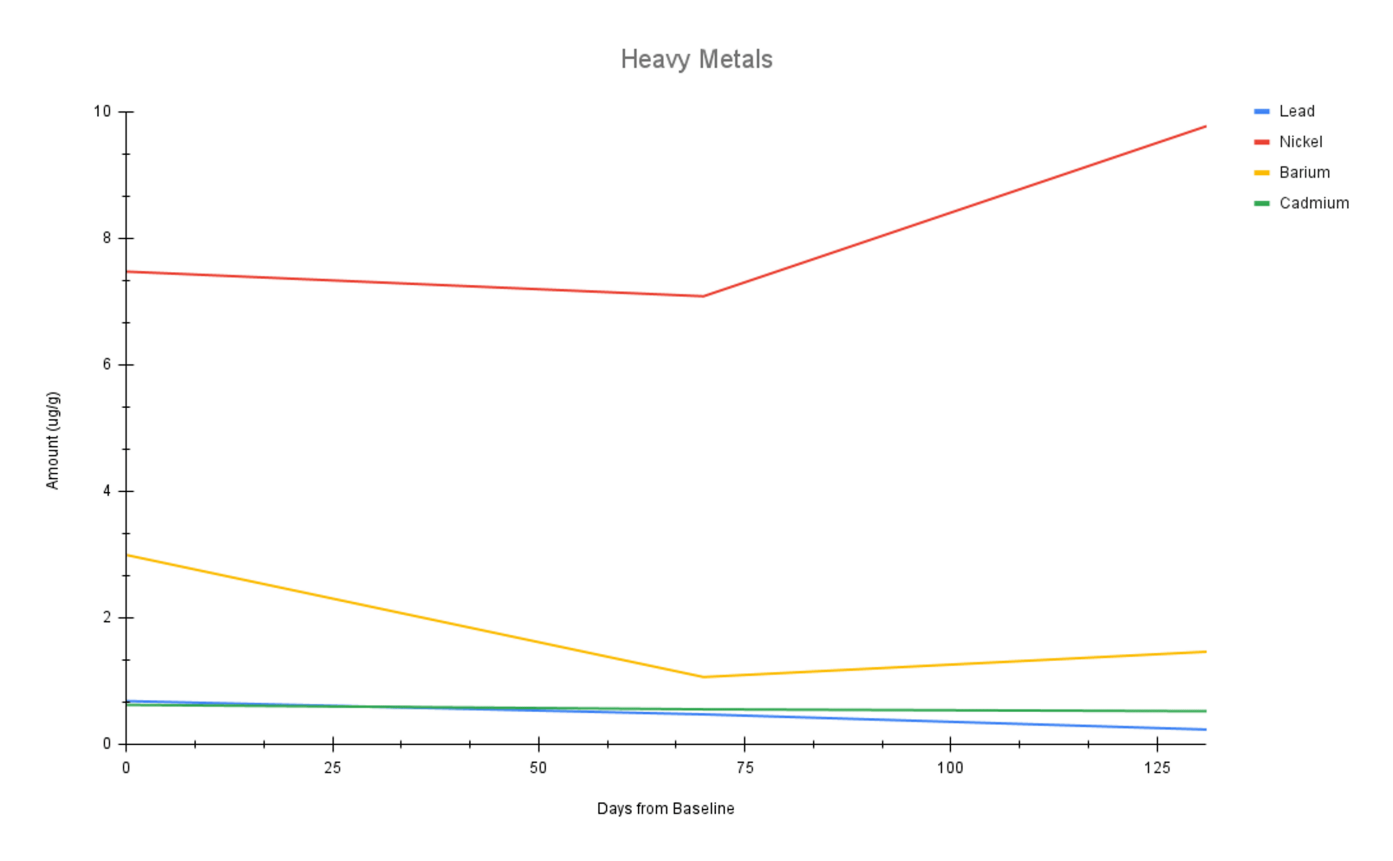
Line chart of the four suboptimal heavy metals over three time points: baseline (day 0), after completion of EBOO series I (day 70), and after completion of EBOO series II (day 131). EBOO: Extracorporeal Blood Oxygenation and Ozonation

**Figure 4:**
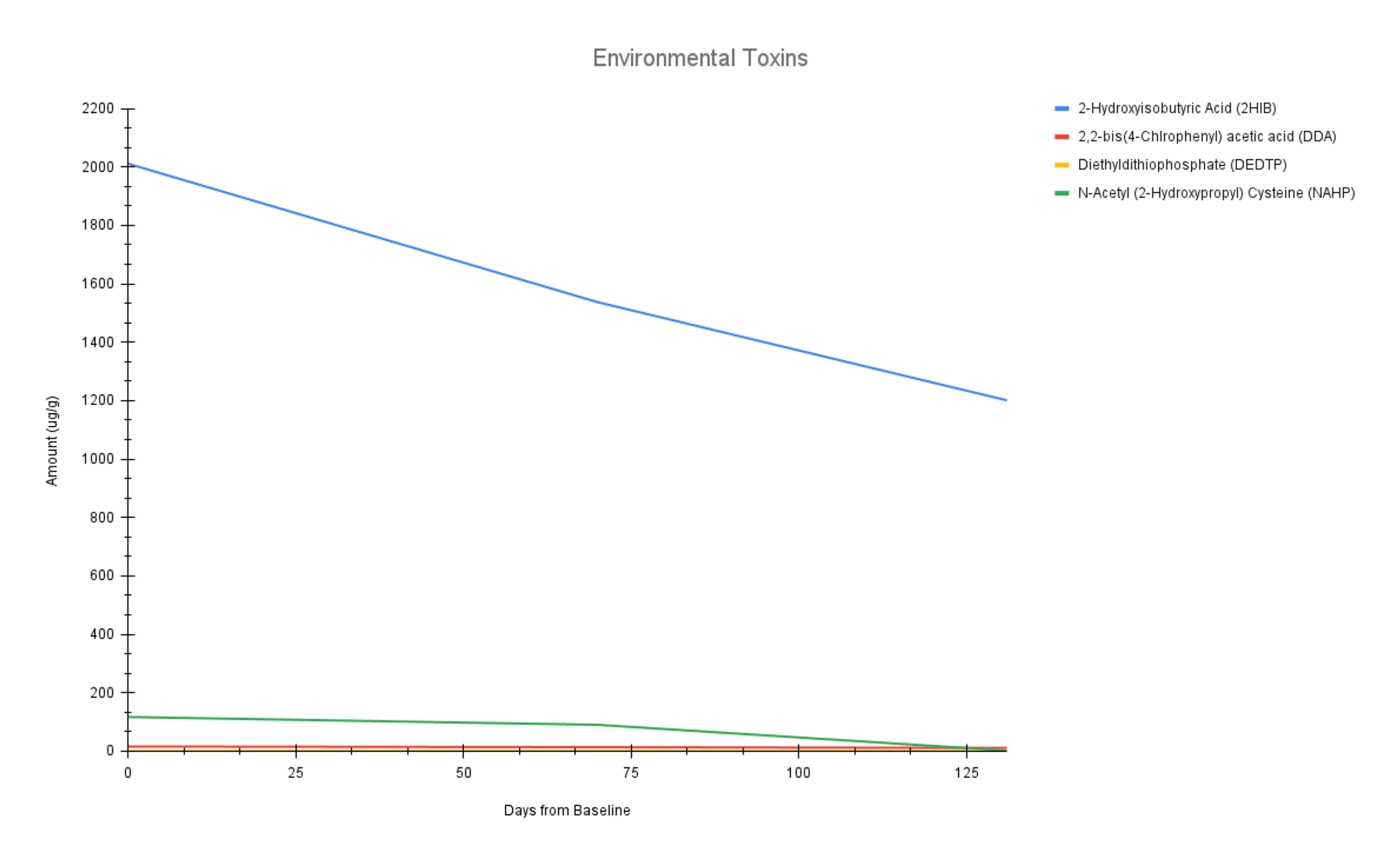
Line chart of the four high and suboptimal environmental toxins over three time points: baseline (day 0), after completion of EBOO series I (day 70), and after completion of EBOO series II (day 131). EBOO: Extracorporeal Blood Oxygenation and Ozonation

Of note, all levels of mycotoxins and environmental toxins decreased over both EBOO series (Figure 2 and Figure 4). Of the heavy metals, lead and cadmium both decreased over both EBOO series, whereas nickel and barium only decreased after completion of EBOO series I (Figure 3). The high and suboptimal toxin raw data can be found in Table 1, as well as their percent decreases across each EBOO series.

**Table 1:** Values of the seven high and suboptimal mycotoxins, four suboptimal heavy metals, and four high and suboptimal environmental toxins at baseline time point 12/19/2025 (day 0), after EBOO series I on 2/27/2025 (day 70), and after completion of EBOO series II on 4/29/2025 (day 131) as well as the toxins’ reference ranges (below 95th percentile). The three rightmost columns show percent decrease of the seven high and suboptimal mycotoxins, four suboptimal heavy metals, and four high and suboptimal environmental toxins from baseline (12/19/2024 or day 0) to after completion of EBOO series I (2/27/2025 or day 70), from completion of EBOO series I (2/27/2025 or day 70) to after completion of EBOO series II (4/29/2025 or day 131), and overall, from baseline (12/19/2024 or day 0) to after completion of EBOO series II (4/29/2025 or day 131). EBOO: Extracorporeal Blood Oxygenation and Ozonation

Toxin Category	Toxin	Reference Range	12/19/25 (Day 0)	2/27/25 (Day 70)	4/29/25 (Day 131)	% Decrease 12/29/24 - 2/27/25	% Decrease 2/27/25 - 4/29/25	% Decrease 12/29/24 - 4/29/25
Mycotoxins (ng/g)	Chaetoglobosin A (CHA)	≤ 31.87	35.64	16.03	3.60	55.02%	77.54%	89.90%
	Enniatin B1 (ENNB1)	≤ 0.22	0.69	0.26	0.15	62.32%	42.31%	78.26%
	Nivalenol (NIV)	≤ 3.2	6.94	3.12	1.07	55.04%	65.71%	84.58%
	Zearalenone (ZEN)	≤ 0.67	1.75	0.75	0.56	57.14%	25.33%	68.00%
	Aflatoxin G1	≤ 6.53	4.27	4.07	2.82	4.68%	30.71%	33.96%
	Aflatoxin M1	≤ 6.4	4.42	3.65	3.62	17.42%	0.82%	18.10%
	Dihydrocitrinone	≤ 16.53	10.76	4.96	2.04	53.90%	58.87%	81.04%
Heavy metals (ug/g)	Lead	≤ 1.16	0.68	0.47	0.23	30.88%	51.06%	66.18%
	Nickel	≤ 12.13	7.47	7.08	9.77	5.22%	-37.99%	-30.79%
	Barium	≤ 5.59	2.99	1.06	1.46	64.55%	-37.74%	51.17%
	Cadmium	≤ 0.8	0.62	0.55	0.52	11.29%	5.45%	16.13%
Environmental toxins (ug/g)	2-hydroxyisobutyric acid (2HIB)	≤ 1215.72	2011.22	1537.00	1200.93	23.58%	21.87%	40.29%
	2,2-bis(4-Chlorophenyl) acetic acid (DDA)	≤ 19	15.72	13.42	10.73	14.63%	20.04%	31.74%
	Diethyldithiophosphate (DEDTP)	≤ 0.3	0.20	0.16	0.10	20.00%	37.50%	50.00%
	N-Acetyl (2-Hydroxypropyl) cysteine (NAHP)	≤ 403	116.70	89.98	1.92	22.90%	97.87%	98.35%

**Figure 5:**
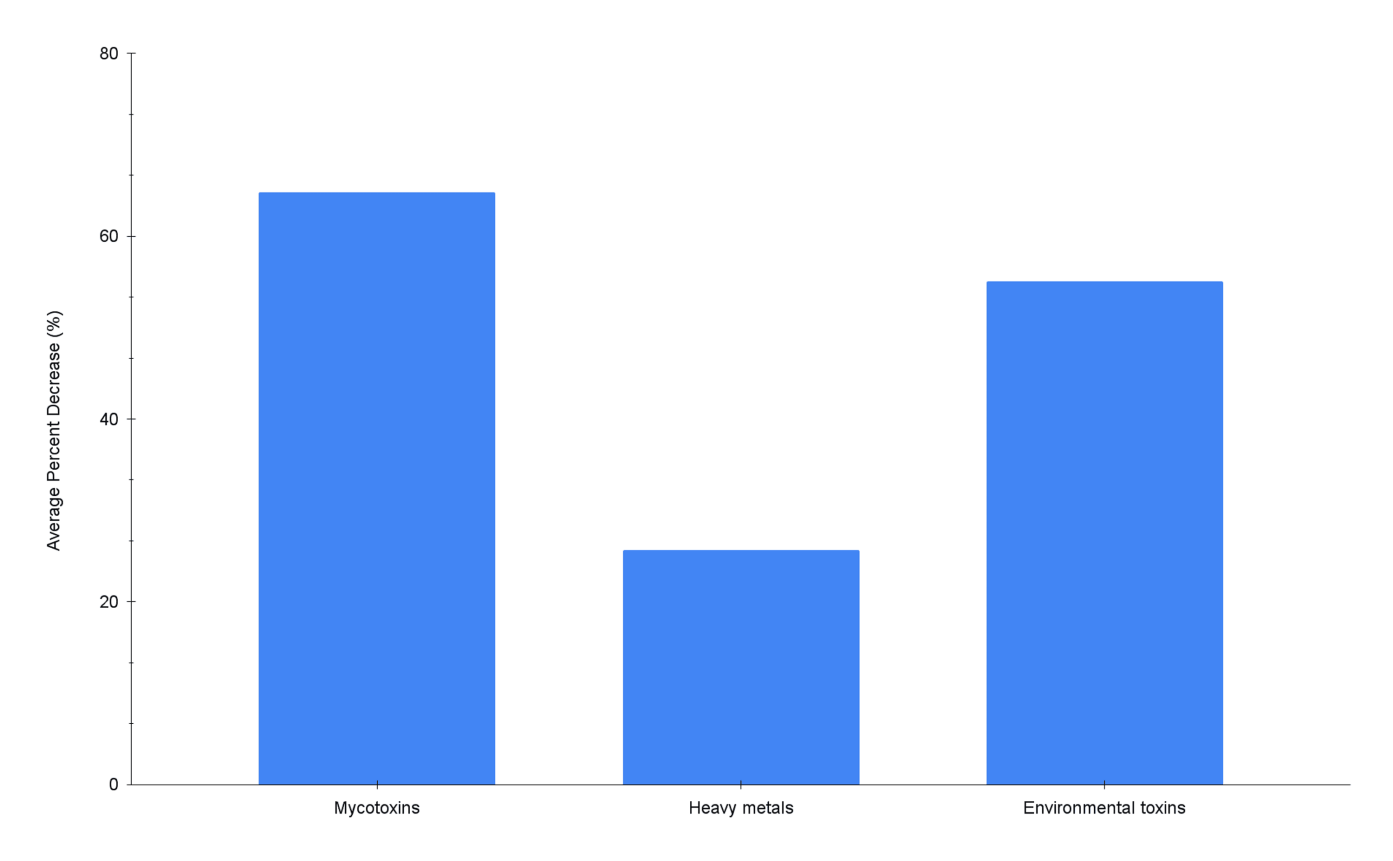
Average percent decrease by toxin class: mycotoxins 64.83%, heavy metals 25.67%, environmental toxins 55.10%.

Additionally, the average decline from baseline to completion of EBOO series II was 64.8% for the mycotoxins, 25.7% for the heavy metals, and 55.1% for the environmental toxins, as depicted in the summary bar chart in Figure 5.

After EBOO series I, the patient’s hemoglobin level was measured at 6.8 g/dL and 7.5 g/dL after EBOO series II. The patient’s baseline hemoglobin level was 7.4 g/dL, but previously had been as low as 4.8 g/dL. This information can be found in Table 2, as well as all other lab values collected, including the raw toxin data included in Table 1.

**Table 2:** Values of all lab work at baseline time point 12/19/2025 (day 0), after EBOO series I on 2/27/2025 (day 70), and after completion of EBOO series II on 4/29/2025 (day 131), as well as their respective reference ranges. EBOO: Extracorporeal Blood Oxygenation and Ozonation; TNP: test not performed

Test Panel	Test Name	Reference Range	12/19/25 (Day 0)	2/27/25 (Day 70)	4/29/25 (Day 131)
Comprehensive metabolic panel (CMP)	Chloride (mmol/L)	98.0 - 107.0	109	109	105
	Sodium (mmol/L)	136.0 - 145.0	144	144	140
	Potassium (mmol/L)	3.5 - 5.1	4.9	4.7	4.3
	Carbon dioxide (mmol/L)	18.0 - 29.0	22	22	23
	Glucose (renal) (mg/dL)	70.0 - 100.0	93	76	94
	BUN (mg/dL)	8.0 - 23.0	13	16	18
	Creatinine (mg/dL)	0.5 - 0.9	0.71	0.81	0.71
	eGFR (mL/min/1.73 m^2^)	≥ 60.0	76.0	64.8	76
	eGFR (African American) (mL/min/1.73 m^2^)	≥ 60.0	88.1	75.2	88
	BUN/creatinine ratio	10.0 - 20.0	18	20	25
	Calcium (mg/dL)	8.9 - 10.6	9.6	8.8	9.2
	Albumin (g/dL)	3.5 - 5.2	4.4	3.7	4.1
	ALT (U/L)	≤ 33.0	12	9	10
	AST (U/L)	≤ 32.0	24	22	23
	Bili, total (mg/dL)	≤ 1.2	0.3	0.3	0.4
	Protein, total (g/dL)	6.2 - 8.0	7.3	6.0	7.1
	Alkaline phosphatase (U/L)	35.0 - 104.0	59	52	65
	Serum osmolality, calculated (mOsm/kg)	285.0 - 315.0	307.6	307.3	300.3
Hormones	SHBG (nmol/L)	17.3 - 125.0	132.0	92.9	110.0
	Testosterone, total (ng/dL)	4.6 - 312.6	2.5	< 2.5	< 2.5
	Dihydrotestosterone (ng/dL)	6.5 - 50.1	4.2	3.4	3.4
	Estradiol (pg/mL)	-	< 10	< 10	< 10
	FSH (mlU/mL)	-	42.9	35.9	34.6
	DHEA-S (μg/dL)	12.0 - 154.0	35.4	26.3	21.4
	LH (mlU/mL)	-	16.9	14.2	16.0
	Free testosterone (ng/dL)	0.03 - 2.62	TNP	TNP	TNP
CBC w/ differential and platelets	WBC (× 10^3^/μL)	3.98 - 10.04	3.89	4.25	4.54
	Hemoglobin (g/dL)	11.2 - 15.7	7.4	6.8	7.5
	Hematocrit (%)	34.1 - 44.9	29.7	24.7	28.2
	MCV (× 10^3^/μL)	79.4 - 94.8	65.7	65.2	67.6
	MCH (pg)	25.6 - 32.2	16.4	17.9	18.0
	MCHC (g/dL)	32.2 - 35.5	24.9	27.5	26.6
	RDW - SD (fL)	36.4 - 46.3	59.1	50.3	48.2
	RDW - CV (%)	11.7 - 14.4	26.0	22.1	20.1
	Lymphocyte count (× 10^3^/μL)	1.18 - 3.74	0.76	1.18	0.76
	RBC (× 10^6^/μL)	3.93 - 5.22	4.52	3.79	4.17
	Platelet count (× 10^3^/ μL)	154.0 - 374.0	368.0	273.0	313.0
	Neutrophils (%)	34.0 - 71.1	68.1	59.3	68.1
	Lymphocytes (%)	19.3 - 51.7	19.5	27.8	16.7
	Monocytes (%)	4.7 - 12.5	8.5	9.6	9.0
	Eosinophils (%)	0.7 - 5.8	1.8	2.6	4.6
	Basophils (%)	0.1 - 1.2	0.80	0.50	0.90
	Immature granulocyte (%)	≤ 2.1	1.3	0.2	0.7
	Neutrophil count (× 10^3^/μL)	1.56 - 6.13	2.65	2.52	3.09
	Monocytes count (× 10^3^/μL)	0.2 - 0.82	0.33	0.41	0.41
	Eosinophil count (× 10^3^/μL)	≤ 0.36	0.07	0.11	0.21
	Basophil count (× 10^3^/μL)	≤ 0.08	0.03	< 0.03	0.04
	Immature granulocyte count (× 10^3^/μL)	≤ 0.1	0.050	< 0.03	< 0.03
	MPV (mean platelet volume) (fL)	9.4 - 12.3	TNP	TNP	TNP
	Nucleated RBC count % (× 10^3^/μL)	≤ 0.012	< 0.01	< 0.01	< 0.01
	Nucleated RBC % (/100WBC)	≤ 0.2	0.0	0.0	0.0
Other markers	Cystatin C (mg/L)	0.61 - 0.95	1.07	1.12	1.22
	Human IGF-1 (ng/mL)	31.0 - 208.0	43	52	43
Anemia	Iron (μg/dl)	37.0 - 145.0	-	-	12
	UIBC (μg/dL)	112.0 - 347.0	-	-	408
	TIBC (μg/dL)	149.0 - 492.0	-	-	420
	Transferrin Saturation (%)	12.0 - 45.0	-	-	< 5
Nutrition	Vitamin D, 25-OH (ng/mL)	30.0 - 108.0	-	-	27.8
Rheumatoid arthritis	hs-CRP	≤ 0.9	0.7	-	-
Cardiac health panel (lipids)	Cholesterol, total (mg/dL)	≤ 199.0	154	148	169
	LDL calculation (mg/dL)	≤ 99.0	72	76	76
	HDL direct (mg/dL)	≥ 66.0	71	64	81
	Cholesterol/HDL ratio	≤ 3.5	2.2	2.3	2.1
	Triglyceride (mg/dL)	≤ 149.0	56	40	58
Cardiac health panel (apolipoproteins)	Apo B (mg/dL)	≤ 89.0	60	72	68
Cardiac health panel (lipoprotein markers)	Lp(a) (mg/dL)	≤ 29.0	< 7	8	< 7
Cardiac health panel (inflammation)	Homocysteine (μmol/L)	≤ 9.0	16	12	14
Glycemic control	Hemoglobin A1c (%)	≤ 5.6	5.4	5.3	5.5
Insulin resistance	Ferritin (ng/mL)	13.0 - 150.0	20	6	7
Thyroid	Free T3 (pg/mL)	2.0 - 4.4	2.7	3.1	2.4
	Free T4 (ng/dL)	0.9 - 1.7	1.1	1.1	1.1
	TSH (μIU/mL)	0.111 - 4.91	2.920	3.380	4.460
	Anti-TPO (IU/mL)	≤ 34.0	< 12	< 12	< 12
	Reverse T3 (ng/dL)	7.0 - 23.0	13	15	12
	Anti-TG (IU/mL)	≤ 115.0	17.1	15.7	15.0
Creatinine	Urine Creatinine (mg/mL)	0.25 - 2.16	1.59	0.49	2.28
Aflatoxins (Mycotoxins)	Aflatoxin B1 (AFB1) (ng/g)	≤ 6.93	3.13	3.50	2.03
	Aflatoxin B2 (AFB2) (ng/g)	≤ 8.13	0.43	3.90	2.57
	Aflatoxin G1 (ng/g)	≤ 6.53	4.27	4.07	2.82
	Aflatoxin G2 (ng/g)	≤ 10.8	4.57	4.47	2.54
	Aflatoxin M1 (ng/g)	≤ 6.4	4.42	3.65	3.62
Other Mycotoxins	Chaetoglobosin A (CHA) (ng/g)	≤ 31.87	35.64	16.03	3.60
	Citrinin (CTN) (ng/g)	≤ 12.53	4.44	4.79	4.34
	Dihydrocitrinone (ng/g)	≤ 16.53	10.76	4.96	2.04
	Enniatin B1(ENN B1) (ng/g)	≤ 0.22	0.69	0.26	0.15
	Fumonisins B1 (ng/g)	≤ 6.13	2.78	2.46	0.57
	Fumonisins B2 (ng/g)	≤ 7.2	0.22	3.72	0.55
	Fumonisins B3 (ng/g)	≤ 10.8	0.25	5.05	0.84
	Gliotoxin (ng/g)	≤ 207.87	43.21	6.81	29.75
	Mycophenolic Acid (ng/g)	≤ 6.4	2.02	1.69	3.05
	Ochratoxin A (OTA) (ng/g)	≤ 6.8	1.74	2.88	3.00
	Patulin (ng/g)	≤ 11.6	5.89	6.46	2.47
	Sterigmatocystin (STC) (ng/g)	≤ 0.53	0.27	0.14	0.22
	Zearalenone (ZEN) (ng/g)	≤ 0.67	1.75	0.75	0.56
Trichothecenes (Mycotoxins)	Deoxynivalenol(DON) (ng/g)	≤ 67.47	20.10	21.81	12.90
	Diacetoxyscirpenol (DAS) (ng/g)	≤ 4.27	1.24	1.04	1.48
	Nivalenol (NIV) (ng/g)	≤ 3.2	6.94	3.12	1.07
	Roridin A (ng/g)	≤ 7.6	1.45	0.17	4.05
	Roridin E (ng/g)	≤ 1.33	0.34	0.59	< 0.05
	Roridin L2 (ng/g)	≤ 6.8	0.49	0.82	0.54
	Satratoxin G (ng/g)	≤ 0.18	0.09	0.06	< 0.05
	Satratoxin H (ng/g)	≤ 0.18	< 0.05	< 0.05	< 0.05
	T-2 Toxin (ng/g)	≤ 0.18	0.05	< 0.05	< 0.05
	Verrucarin A (ng/g)	≤ 1.33	0.31	0.35	0.25
	Verrucarin J (ng/g)	≤ 9.2	0.53	1.48	1.15
Heavy metals (urine)	Aluminum (μg/g)	≤ 45.15	14.25	8.01	< 3
	Antimony (μg/g)	≤ 0.16	0.06	< 0.02	< 0.02
	Arsenic (μg/g)	≤ 52	11.29	7.59	9.76
	Barium (μg/g)	≤ 5.59	2.99	1.06	1.46
	Beryllium (μg/g)	≤ 0.76	< 0.1	0.11	< 0.1
	Bismuth (μg/g)	≤ 2.53	< 0.1	< 0.1	< 0.1
	Cadmium (μg/g)	≤ 0.8	0.62	0.55	0.52
	Cesium (μg/g)	≤ 10.3	5.96	3.48	3.37
	Gadolinium (μg/g)	≤ 0.45	< 0.05	< 0.05	< 0.05
	Lead (μg/g)	≤ 1.16	0.68	0.47	0.23
	Mercury (μg/g)	≤ 1.61	< 0.1	< 0.1	0.14
	Nickel (μg/g)	≤ 12.13	7.47	7.08	9.77
	Palladium (μg/g)	≤ 0.2	< 0.1	< 0.1	< 0.1
	Platinum (μg/g)	≤ 0.9	< 0.05	< 0.05	< 0.05
	Tellurium (μg/g)	≤ 0.89	< 0.03	0.17	0.07
	Thallium (μg/g)	≤ 0.43	0.18	0.22	0.12
	Thorium (μg/g)	≤ 0.07	< 0.01	< 0.01	< 0.01
	Tin (μg/g)	≤ 3.72	0.22	< 0.2	0.28
	Tungsten (μg/g)	≤ 0.33	< 0.04	0.04	0.10
	Uranium (μg/g)	≤ 0.04	< 0.01	< 0.01	< 0.01
Environmental phenols (environmental toxins)	4-Nonylphenol (μg/g)	≤ 2.06	0.24	0.03	0.19
	Bisphenol A (BPA) (μg/g)	≤ 5.09	0.21	1.37	0.44
	Triclosan (TCS) (μg/g)	≤ 358	1.96	10.37	20.57
Herbicides (Environmental toxins)	2,4-dichlorophenoxyacetic acid (2,4-D) (μg/g)	≤ 1.55	0.19	0.44	0.28
	Atrazine (μg/g)	≤ 0.05	0.01	< 0.01	0.02
	Atrazine mercapturate (μg/g)	≤ 0.05	0.01	0.01	0.01
	Glyphosate (μg/g)	≤ 7.6	0.68	0.74	1.22
Mitochondrial Marker (Environmental toxins)	Tiglylglycine (TG) (μg/g)	≤ 3.24	0.03	0.08	0.02
Other markers (Environmental toxins)	Diphenyl Phosphate (DPP) (μg/g)	≤ 3.7	0.10	0.62	0.80
	N-acetyl-S-(2-carbamoylethyl)-cysteine (μg/g)	≤ 199	0.25	8.82	4.51
	Perchlorate (PERC) (μg/g)	≤ 10.7	1.12	4.32	0.04
Parabens (Environmental toxins)	Butylparaben (μg/g)	≤ 4.39	0.20	0.12	0.04
	Ethylparaben (μg/g)	≤ 99.3	0.50	0.18	0.05
	Methylparaben (μg/g)	≤ 653	0.21	169.03	0.11
	Propylparaben (μg/g)	≤ 222	0.02	0.11	0.19
Pesticides (Environmental toxins)	2,2-bis(4-Chlorophenyl) acetic acid (DDA) (μg/g)	≤ 19	15.72	13.42	10.73
	3-Phenoxybenzoic Acid (3PBA) (μg/g)	≤ 5.44	0.26	0.29	0.34
	Diethyl phosphate (DEP) (μg/g)	≤ 15.7	0.04	0.19	2.23
	Diethyldithiophosphate (DEDTP) (μg/g)	≤ 0.3	0.20	0.16	0.10
	Diethylthiophosphate (DETP) (μg/g)	≤ 3.92	0.11	1.10	0.05
	Dimethyl phosphate (DMP) (μg/g)	≤ 33.6	2.34	4.63	1.30
	Dimethyldithiophosphate (DMDTP) (μg/g)	≤ 6.12	0.22	0.60	0.57
	Dimethylthiophosphate (DMTP) (μg/g)	≤ 33.7	3.40	4.64	3.82
Phthalates (Environmental toxins)	Mono-(2-ethyl-5-hydroxyhexyl) phthalate (MEHHP) (μg/g)	≤ 37.7	2.38	0.11	12.04
	Mono-(2-ethyl-5-oxohexyl) phthalate (MEOHP) (μg/g)	≤ 23.4	2.23	1.21	6.05
	Mono-2-ethylhexyl phthalate (MEHP) (μg/g)	≤ 8.47	0.95	0.02	0.33
	Mono-ethyl phthalate (MEtP) (μg/g)	≤ 541	4.59	14.42	0.56
Volatile organic compounds (Environmental toxins)	2-hydroxyethyl mercapturic acid (HEMA) (μg/g)	≤ 4.75	0.27	0.10	0.04
	2-hydroxyisobutyric acid (2HIB) (μg/g)	≤ 1215.72	2011.22	1537.00	1200.93
	2-methylhippuric acid (2MHA) (μg/g)	≤ 248	4.56	36.56	13.95
	3-methylhippuric acid (3MHA) (μg/g)	≤ 612.83	0.96	5.66	5.53
	4-methylhippuric acid (4MHA) (ug/g)	≤ 752.72	0.93	33.13	8.67
	N-acetyl (2-Cyanoethyl) Cysteine (NACE) (μg/g)	≤ 256	0.93	1.64	0.10
	N-acetyl (2, Hydroxypropyl) Cysteine (NAHP) (μg/g)	≤ 403	116.70	89.98	1.92
	N-acetyl (3,4-Dihydroxybutyl) Cysteine (μg/g)	≤ 583	0.02	0.26	2.10
	N-Acetyl (Propyl) Cysteine (NAPR) (μg/g)	≤ 46.1	1.08	4.66	0.39
	N-acetyl phenyl cysteine (NAP) (μg/g)	≤ 3.03	0.05	0.16	0.19
	Phenyl glyoxylic Acid (PGO) (μg/g)	≤ 518	60.10	239.79	11.09

## Discussion

Despite its growth and ubiquity in the population, mycotoxin, heavy metal, and environmental toxicity remain a therapeutic challenge. This case highlights the potential use of EBOO as a means of removing these toxins in a patient suffering from anemia. Anemia, which affects around 30% of reproductive-age women worldwide [42], as well as many other chronic conditions, may be secondary to environmental toxicities. Thus, by treating elevated toxin levels, EBOO may serve as a potential mediator for long-term diseases and disorders.

One study utilizing a mouse model showed that glutathione and other antioxidants were elevated in the livers of mice that were fed maize containing mycotoxins, indicating the presence of oxidative stress with a mycotoxin-contaminated diet [43]. Similarly, many heavy metals, such as nickel and cadmium, deplete glutathione, contributing to the generation of reactive oxygen and nitrogen species [44]. Thus, ozonation of the blood ex vivo, such as by EBOO, may help to trigger the immune system to respond to high levels of toxins in the body once the blood has been returned to the patient.

Though the underlying mechanism behind EBOO’s effectiveness in reducing toxin levels cannot be established from a single case, this case report demonstrates promising results that EBOO can serve as a treatment for toxicities such as mycotoxicity, heavy metal toxicity, and various environmental toxicities. As such, all of the patient’s levels decreased from baseline to day 70 as well as from baseline to day 131, with two exceptions. Though barium decreased overall with a percent decrease of 51.17%, it increased from the completion of EBOO series I to EBOO series II. Additionally, nickel was the only toxin that showed an overall increase from baseline to completion of EBOO series II. While barium plays no known role in biological functions [45], nickel plays a role in iron metabolism. Nickel interferes with iron homeostasis in multiple ways; high levels are often seen in cases of anemia [46-47]. It is unknown if the patient experienced any environmental or dietary changes that would have triggered this increase in nickel levels, though it is possible that her elevated nickel levels contributed to her low iron levels. We are unsure if there were any changes to the patient’s diet or environment at this time. Additionally, as per the manufacturer, all tubing and materials are nickel-free; thus, a rise in nickel levels potentially reflects re-exposure or mobilization.

Due to the human body’s ability to break down toxins primarily through hepatic metabolism and renal excretion, it is within reason to assume that the levels of these toxins would naturally decrease over time. However, because of the ubiquity of these toxins, primarily in food and the environment, it is most likely that the patient experienced constant exposure, especially considering the information regarding the old building she lived in. The rate of decay of mycotoxins, heavy metals, and environmental toxins is unknown, assuming constant environmental exposure. Therefore, to build a stronger evidence base, it is essential to collect more data on the efficacy of EBOO from a large sample size.

While the patient’s toxin levels displayed promising declines, it is of note that her hemoglobin levels fluctuated within a small range. Her baseline hemoglobin, 7.4 g/dL, decreased to 6.8 g/dL after completion of EBOO series I and then increased to 7.5 g/dL after completion of EBOO series II. Thus, there was no clinically meaningful change in hemoglobin levels despite the 64.8% average decrease for the mycotoxins, the 25.7% average decrease for the heavy metals, and the 55.1% average decrease for the environmental toxins. Despite her slight decline in hemoglobin levels of EBOO series I, the patient reported feeling slightly better at that time. Interestingly, as her hemoglobin levels increased back to baseline after completion of EBOO series II, she reported feeling worse. Though this study focuses on the use of EBOO as a treatment for high toxin levels, it is useful to see how these toxin levels contribute to chronic conditions. However, it is unknown how well the patient adhered to her prescribed iron regimen. Additionally, because this is a single report, monitoring the patient’s levels at follow-up appointments can provide more information as to whether the EBOO is the source of the large decline in toxin levels.

## Conclusions

The purpose of this case report is to document the use of EBOO as a treatment for removing mycotoxins, heavy metals, and environmental toxins in an 88-year-old female experiencing chronic anemia. Over two series of three EBOO procedures, all of her toxin levels decreased, with the exception of nickel. To our knowledge, our case serves as the first of its kind to report direct measurements of toxins before and after EBOO. This case underscores the importance of elevated toxin levels as a mediator in anemia, a chronic condition. Future large-scale studies are necessary to clarify predictive factors and guide appropriate interventions for prevalent toxicities.
